# A small molecule, C_24_H_17_ClN_4_O_2_S, inhibits the function of the type III secretion system in *Salmonella* Typhimurium

**DOI:** 10.1186/s43141-022-00336-1

**Published:** 2022-04-05

**Authors:** Rerngwit Boonyom, Sittiruk Roytrakul, Patipat Thinwang

**Affiliations:** 1 Department of Medical Technology, Faculty of Allied Health Sciences, Naresuan University, Phitsanulok, 65000 Thailand; 2 Proteomics Research Laboratory, National Center for Genetic Engineering and Biotechnology, National Science and Technology Development Agency, Pathum Thani, 12120 Thailand

**Keywords:** Type III secretion system (T3SS), T3SS inhibitor, *Salmonella* pathogenicity island 1 (SPI-1), InvC ATPase

## Abstract

**Background:**

*Salmonella enterica* serovar Typhimurium (*S*. Typhimurium) causes gastroenteritis and diarrhea in humans and food-producing animals. The type III secretion system (T3SS) has been known to be a potent virulence mechanism by injecting effector proteins into the cytosol of host cells. *S*. Typhimurium encodes two T3SSs by *Salmonella* pathogenicity islands 1 and 2. Previous studies showed that T3SS shared a potent virulence mechanism and molecular structure among several gram-negative bacteria. Therefore, T3SS has been identified as an attractive target in the development of novel therapeutics for the treatment of bacterial infections. Several studies reported that small-molecule compounds are able to inhibit functions of bacterial T3SSs. A small molecule, C_24_H_17_ClN_4_O_2_S, has been shown the ability to inhibit the activity of *Yersinia pestis* T3SS ATPase, YscN, resulting to block the secretion of effector proteins. In this study, we studied the effects and mechanism for SPI-1 T3SS inhibition of this compound in *S.* Typhimurium.

**Results:**

We demonstrated that this compound prohibited the secretion of effector proteins from *Salmonella via* SPI-1 T3SS at 100 μM. As the result, bacterial invasion ability into epithelial cell cultures was reduced. In contrast with previous study, the C_24_H_17_ClN_4_O_2_S molecule did not inactivate the activity of SPI-1 T3SS ATPase, InvC, in *Salmonella*. However, we studied the global cellular effects of *S*. Typhimurium after being treated with this compound using a quantitative proteomic technique. These proteomic results showed that the main SPI-1 transcription regulator, InvF, and two effector proteins, SipA and SipC, were reduced in bacterial cells treated with the compound.

**Conclusions:**

It may explain that action of the small-molecule compound, C_24_H_17_ClN_4_O_2_S, for blocking the secretion of SPI-1 T3SS in *Salmonella* is through inhibition of SPI-1 regulator, InvF, expression. Further studies are necessary to identify specific mechanisms for inhibition between this small-compound and InvF SPI-1 regulator protein.

## Background

Foodborne infections caused by contamination of *Salmonella* spp. in food products. Pre-slaughter pigs and chickens have been incriminated as the major sources of *Salmonella* contaminations in chickens and pork products at later stages in the food processing chain [1]. The genus *Salmonella* is composed of motile bacteria that belong to the family Enterobacteriaceae: facultative anaerobic gram-negative bacilli, 2 to 3 μm × 0.4 to 0.6 μm in size. There are two main clinical diseases associated with *Salmonella* infection: the systemic disease typhoid fever is caused by *Salmonella enterica* serovar Typhi (*S*. Typhi) and non-typhoidal salmonellosis also known as enteritis [2]. *S.* Typhimurium commonly causes disease in animals, and it can be generally transmitted to humans via food derived from an infected livestock. In humans, the infection manifests as an intestinal disease ranging from gastroenteritis, usually lasting 3–5 days, to a more debilitating enteritis lasting 2–3 weeks [3].

For molecular pathogenesis of *S.* Typhimurium, bacteria contain two virulence-associated gene clusters, *Salmonella* pathogenicity islands 1 and 2 (SPI-1 and SPI-2). Both of these SPIs encode type III secretion systems (T3SSs) [4]. The function of two T3SSs is the translocation of virulence factors or effector proteins from the bacterial cytoplasm into the host cell, functioning as “molecular syringes” [5]. SPI-1 and SPI-2 express at different times during infection. Whereas the SPI-1 is required for the initial interaction of *Salmonella* with intestinal epithelial cells, the SPI-2 encoded system is required for systemic infection. The SPI-1 has been shown to be an essential factor for invasion in non-phagocytic cells, induction of intestinal inflammatory responses and diarrhea, as well as colonization of the intestine [6]. Contrasting, the SPI-2 has an important role in bacterial survival in macrophages [7]. At least 13 effector proteins are delivered by the SPI-1 T3SS. The translocated effectors alter key host-cell functions including signal transduction, cytoskeletal architecture, membrane trafficking, and cytokine expression. These processes command host cells to engulf bacteria into the cytosol by bacterial-mediated endocytosis or macropinocytosis [8]. After entering host cells, bacterial SPI-2 T3SS are expressed and secreted SPI-2 effector proteins for forming the large *Salmonella* containing vacuoles (SCVs) surrounding bacterial cells [9]. Moreover, the function of SPI-2 effectors is essential for bacterial replication in systemic infection [10].

The T3S mechanism has been described by Akeda et al. [11]. The pore size of T3SS needle is estimated about 28 A°. Therefore, the chaperone-effector complex must be separated before translocation. The chaperone and effector protein are unfolded by T3SS ATPase located in the basement of T3S machinery [11, 12]. In *S.* Typhimurium, T3SS ATPase, InvC, is able to recognize both chaperone and complex. Before T3S effector secretion, the chaperone-effector complex binds with InvC ATPase. Then, the InvC enzyme releases chaperone from its cognate substrate and induces the global unfolding of effector protein. Finally, the unfolding effector is secreted through the central channel of the T3SS-associated needle complex [11]. The previous study suggested that the proton motive force (PMF) has been involved in the secretion of proteins through T3SS [13].

There is an increase in antibiotic-resistant bacterial strains. It will be a crucial factor to develop new antimicrobial agents. The discovery of new drugs that specifically target pathogenic properties without killing bacteria might decrease the chance of bacterial resistance emerging against drugs. Therefore, the T3SS is an attractive target for new anti-bacterial drugs, because T3SSs are primary virulence mechanisms in pathogenic gram-negative bacteria and are not required for bacterial survival [14]. Moreover, these systems are conserved in a variety of gram-negative bacteria [15]. Previous studies that showed small molecules could inhibit T3SS functions in gram-negative bacteria [16–19]. Some small molecules prevent the secretion of the effector protein from *Yersinia pestis* (*Y. pestis*) into bacterial medium and mammalian cells. The docking conformational model indicated that these molecules tightly bind inside the hydrophobic clave of YscN ATPase of *Y. pestis* resulting in loss of enzyme function [17]. In addition, this study showed that *N*-[2-[4-(benzimidazol-1-yl)anilino]-2-oxoethyl]-3-chloro-1-benzothiophene-2-carboxamide, C_24_H_17_ClN_4_O_2_S, the compound was capable of inhibiting both effector protein secretion and ATPase activity in *Y. pestis*. Moreover, this compound was also non-toxic to the mammalian cells due to a low potential to target human enzymes [17]. We hypothesized that the C_24_H_17_ClN_4_O_2_S compound also has inhibitory effects on InvC ATPase in *S.* Typhimurium. However, we found that the C_24_H_17_ClN_4_O_2_S compound did not show ATPase inhibitor activity in *S.* Typhimurium, but this compound prohibited the expression of the main SPI-1 transcription regulator, InvF. We hope that results from this study may lead to develop novel therapeutics.

## Methods

### The small molecule

**Fig. 1:**
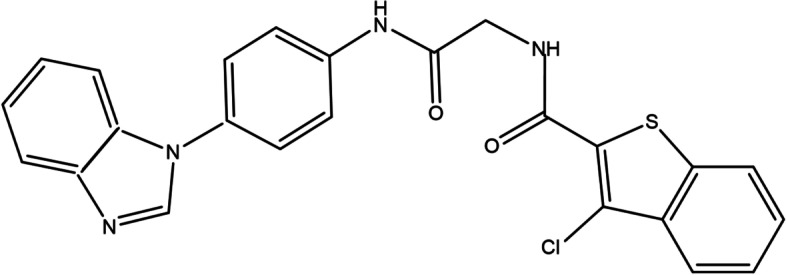
Structure of C_24_H_17_ClN_4_O_2_S compound

The *N*-[2-[4-(benzimidazol-1-yl)anilino]-2-oxoethyl]-3-chloro-1-benzothiophene-2-carboxamide, C_24_H_17_ClN_4_O_2_S, compound (Fig. 1) was purchased from commercial vendor Enamine and Life Chemicals (USA).

### Cell line and bacterial cell culture conditions

Cell lines, bacterial strains, and plasmids used in this study are listed in Table 1. The human colon adenocarcinoma cell line, HT29, is maintained in DMEM containing 10% FBS.

**Table 1:** Cell line, bacterial strains, and plasmids used in this study

Cell line, bacterial strain or plasmid	Properties	Reference or source
Cell line		
HT29	Human colon adenocarcinoma cell line	ATCC
*Salmonella* Typhimurium strains		
SL1344	Wild-type	*Salmonella* genetic stock center
SA	*sipA*::strep-tag	Current study
SW002	*invC::kan*^R^	[20]
*Escherichia coli* strains		
BL21 (DE3)	F–, *omp*T, *hsd*SB (rB–, mB–), *gal*, *dcm* (DE3)	Novagen
Plasmids		
pJBT	pBAD24 derivative with C-terminal strep-tag	[21]
pJWA	pJBT derivertive with full-length SipA	Current study
pJWAK	pJWA derivative containing kanamycin resistance gene	Current study
pKD13	Template plasmid for kanamycin cassette	[22]
pKD46	Red recombinase expression plasmid	[22]
pCP20	Flp recombinase expression plasmid	[22]
pGEX-2T pGWC	Expression vector with GST fusion pGEX-2T derivative with full-length InvC	GE Healthcare Current study

*S.* Typhimurium SL1344 and isogenic mutants were grown in Luria-Bertani (LB) medium overnight and diluted 1:100 into the fresh medium with 0.3 M NaCl, SPI-1 induction condition [23]. Bacterial cells were grown for 4 h at 37 °C, and 200 rpm. *E. coli* strain BL21 (DE3) was used for protein overexpression. *E. coli* was grown in LB medium containing appropriate antibiotics at 37 °C and 200 rpm. The expression of recombinant protein was induced with 0.5 mM IPTG and left for 3 h at 37 °C.

### Plasmids and strep-tag chromosomal tagging in SL1344 constructions

All recombinant DNA techniques in this study were based on standard protocols [24]. The DNA primers were synthesized by Biobasic, Canada. The primers used in this work are listed in Table 2. The PCRs were carried out using Phusion^TM^ DNA Polymerase (Finnzymes, USA), and ligations were performed using T4 DNA ligase (Fermentas, USA).

**Table 2:** Primer sets used in this study

Primer set	Forward primer sequence (5′-3′)	Reverse primer sequence (5′-3′)
CF	GCGTggatccAAAACACCTCGTTTACTGCA ATATC	GATgaattcTTAATTCTGGTCAGCGAATGCATT CATA
AF	AGgaattcACCATGGTTACAAGTGTAAGGA CTCAGCC	TCGAaagcttACGCTGCATGTGCAAGCCATCAA C
KFR	GAGAgaagattttcAGATTGCAGCATTACA CGTCTTGAGC	TATGAgaattaattcCGGGGATCCGTCGACCTG CAGTTCG
SA	GGCGAGCTGGCCCGGCTTACGAGTC	**TTGCTTCAATATCCATATTCATCGCATCTTT** **CCCGGTTA***AATTCCGGGGATCCGTCG*

Lower case type indicates an engineered restriction enzyme site

Underlined-case type indicates complementary sequences to *Salmonella sipA* gene loci

Bold case type indicates complementary sequences to nucleotide sequences downstream *sipA* gene loci

Italic case type indicates complementary sequences to *kan*^R^ gene

A set of CF primers was used to amplify a full length of the InvC-coding region. The resulting product was digested with *BamH*I and *EcoR*I and cloned into *BamH*I and *EcoR*I-digested pGEX-2T, giving pGWC. This recombinant plasmid expressed the GST-InvC fusion protein under the control of tac promoter.

For the construction of strep-tag chromosomal tagging in the SL1344 strain, the amplification of DNA fragments encoding the full length of SipA effector was conducted by PCR using a set of AF primers. The PCR products were digested with *EcoR*I and *Hind*III and cloned into pJBT by utilizing the same restriction enzymes to produce a pJWA recombinant plasmid. This recombinant plasmid was expressed by the SipA protein fused with a C-terminal strep-tag under control of the inducible arabinose promotor of pBAD24. For the construction of SL1344 containing strep-tag chromosomal tagging strain, DNA fragments encoding kanamycin-resistant gene were amplified using pKD13 as a template and a set of KFR primer. The PCR products were digested with *Xmn*I and cloned into pJWA by utilizing the same restriction enzyme to produce pJWAK plasmid. This vector containing kanamycin-resistant gene (*kan*^R^) was downstream from DNA fragment encoding SipA effector protein. The recombinant plasmids were verified by restriction analysis and DNA sequencing (Macrogen, South Korea).

*S.* Typhimurium SA strain was engineered from the SL1344 strain. This strain was chromosomally in-fusion between DNA fragment encoding strep-tag epitope on C-terminus of SipA effector protein using λ Red recombination system as described by Datsenko and Wanner [22]. Epitope fusion cassettes were amplified using pJWAK plasmid as a template and a set of SA primers. DNA fragments of epitope fusion cassettes were purified and used for electroporation into *S.* Typhimurium SL1344 harboring pKD46 for expression of Red recombinase. Kanamycin-resistant transformants were selected at 37 °C. Then, the kanamycin-resistant gene was eliminated after the transformation of pCP20. The proper mutant strains were confirmed by PCR and verified by DNA sequencing (Macrogen, South Korea).

### Blocking of effector protein secretion in a bacterial cell culture

*S.* Typhimurium SL1344 or SA strains were grown in SPI-1 induction condition, as described previously. The small-molecule compound was added to the culture medium at various concentrations. Culture of *S.* Typhimurium strain SW002, a T3S-defective (*invC*::*kan*^R^) was used as a positive control. A negative control added DMSO without compound. Bacterial cells and culture supernatants were separated by centrifugation at 6000 g for 10 min. The supernatants were filter-sterilized (0.22- μm pore size), and proteins were precipitated with 10% TCA and acetone. The precipitated sample was separated on SDS-PAGE and stained in Coomassie blue stain solution. For detection of SipA strep-tag fusion protein, supernatant from *S.* Typhimurium SA strain was electrophoresed on SDS-PAGE and analyzed by Western blotting with polyclonal antibody against strep-tag (IBA Lifesciences, Germany).

### Inhibition of bacterial invasion

The cell line HT29 approximately 2 × 10^4^ cells were seeded in 96-well culture plate supplement with DMEM medium for overnight. HT29 cells were infected with untreated or treated bacteria at a MOI of 100. After incubation of 15 min to allow bacterial entry into cells, cells were washed with PBS and DMEM containing 100 μg/mL of gentamicin for killing any remaining extracellular bacteria. After washing, cells were lysed with solubilizing buffer. The number of intracellular bacteria was calculated by plating on LB agar. *S.* Typhimurium SW002 strain was used as a positive control, while bacteria SL1344 strain treated with DMSO was served as a negative control.

### Recombinant protein expression and purification

*E. coli* BL21 (DE3) cells harboring pGWC recombinant plasmids were grown in LB medium with ampicillin (100 μg/mL) at 37 °C to OD_600_ ~0.4, induced with 0.5 mM IPTG and incubated at 37 °C for an additional 4 h. Bacterial cells were collected by centrifugation (6000 g, 10 min) and stored at −80 °C.

For the protein purification step, bacterial cell pellets were resuspended in pre-chilled PBS buffer supplemented with a complete protease inhibitor cocktail (Sigma Aldrich, USA). The bacterial suspension was homogenized by sonication. The soluble fraction containing GST-InvC fusion proteins was passed through 0.45-μm pore size filter. The bacterial clear lysate was mixed with pre-washed Glutathione-Agarose resins (Genscript, USA) and incubated for 30 min at 4 °C. Glutathione beads bound with GST-InvC proteins were washed three times with PBS plus 0.1% Tween 20. The GST-InvC recombinant proteins were eluted with PBS containing 10-mM glutathione. The eluted fraction was analyzed by SDS-PAGE. The total protein concentration was determined using the Bradford protein assay.

### ATPase enzyme activity assay

The ATPase enzyme activity of purified InvC recombinant protein was determined using ADP-Glo^TM^ MAX Assay (Promega, USA). This kit is to monitor the activity of ADP-generating enzymes. The principle is the production of ADP from ATP by ATPase. After that, reagents in this kit reconvert ADP to ATP and measurement by luciferase/luciferin reaction. The methods of an experiment performed according to the manufacturer’s instructions. The small molecule compound was added to the ATPase reaction at various concentrations. The light generated from the luciferase/luciferin reaction indicates ATPase activity. The reaction with DMSO was used as a control.

### Bacterial cell proteomic analysis by GeLC-MS

Bacterial lysate from treated or untreated with the small compound was separated on SDS-PAGE. After protein bands were excised, the gel plugs were dehydrated with 100% acetonitrite with 10-mM DTT in 10-mM ammonium bicarbonate at room temperature for 1 h and alkylated for 1 h in room temperature for dark in presence of 100-mM iodoacetamide in 10-mM ammonium bicarbonate. After alkylation, the gel pieces were dehydrated twice with 100% ACN for 5 min. To perform in-gel digestion of proteins, 10 μL of trypsin solution (10 ng/μl trypsin in 50% ACN/10mM ammonium bicarbonate) was added to the gels followed by incubation at room temperature for 20 min, and then, 20 μl of 30% ACN was added to keep the gels immersed throughout digestion. The gels were incubated at 37 °C for a few hours or overnight. To extract peptide digestion products, 30 μl of 50% ACN in 0.1% formic acid (FA) was added into the gels, and then, the gels were incubated at room temperature for 10 min in a shaker. Peptides extracted were collected and pooled together in the new tube. The pool extracted peptides were dried by vacuum centrifuge and kept at −80 °C for further mass spectrometric analysis.

LC-MS/MS analysis of digested peptide mixtures was performed using a Waters SYNAPT™ HDMS™ system. The 1D-nanoLC was carried out with a Waters nanoACQUITY UPLC system. Four μL of tryptic digests were injected onto the RP analytical column (20 cm × 75 μm) packed with a 1.7-μm bridged ethyl hybrid (BEH) C18 material (waters). Peptides were eluted with a linear gradient from 2 to 40% acetonitrile developed over 60 min at a flow rate of 350 nL/min. This was followed by a 15-min period of 80% acetonitrile to clean the column before returning to 2% acetonitrile for the next sample. The effluent samples were electrosprayed into a mass spectrometer (Synapt HDMS) for MS/MS analysis of peptides and then generated the spectral data for further protein identification against database search.

Mass lists in the form of Mascot generic files were created and used as the input for Mascot MS/MS Ions searches of the National Center for Biotechnology Information non-redundant (NCBInr) database (www.matrixscience.com). Default search parameters used were the following: enzyme = trypsin, max. missed cleavages =1; fixed modifications = carbamidomethyl (C); variable modifications = oxidation (M); peptide tolerance ±1.2 Da; MS/MS tolerance ±0.6 Da; peptide charge = 1+, 2+, and 3+; and instrument = ESI-QUAD-TOF.

### Statistical analysis

All statistical analyses were performed using GraphPad Prism 7 (GraphPad Software, San Diego, CA). The data were presented as mean and standard deviation (SD). Student *t* test was used to consider the statistical significance at a *P* value of <0.05.

## Results

Small molecule, C_24_H_17_ClN_4_O_2_S, was concentration-dependent inhibition of SPI-1 T3S effector proteins but did not affect the growth of *S.* Typhimurium

**Fig. 2:**
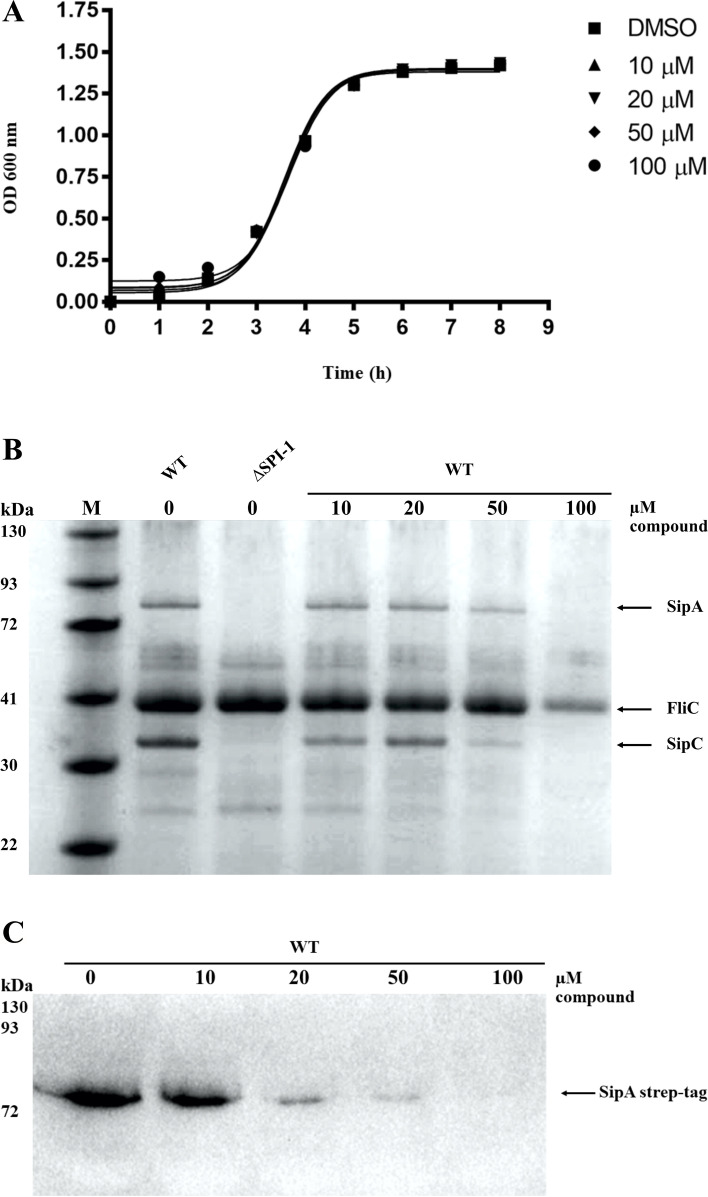
Concentration of compound affected effector protein secretion through T3SS but did not interfere bacterial growth. **A** Growth curves of *S.* Typhimurium at various concentration of compound. The control was bacterial culture with DMSO. The results from SDS-PAGE (**B**) and Western blotting (**C**) indicated that at 100 μM of compound inhibited T3S. The SPI-1 deficiency strain (*invC::kan*^*R*^) was used as control

Firstly, we investigated the effect of this compound on bacterial growth. Bacteria were grown in media in presence of a small molecule, C_24_H_17_ClN_4_O_2_S, at concentrations of 10, 20, 50, and 100 μM for 8 h. The results showed that there was no difference between bacterial growth rates in any concentrations of small-compound and DMSO control (Fig. 2A). For SPI-1 T3S inhibition, the bacterial cell was grown in LB medium with 0.3 M NaCl for SPI-1 expression condition supplement with compounds at concentrations of 10, 20, 50, and 100 μM. The total protein in bacterial supernatants was precipitated and analyzed by SDS-PAGE. Protein profiles show that the compound exhibited an inhibitory effect on the secretion of SPI-1 effector proteins (SipA and SipC) at a concentration of 100 μM. This compound inhibited the secretion of SipA and SipC in a dose-dependent manner. Additionally, 100 μM of this concentration has an evident effect on flagellar protein FliC (Fig. 2B). For confirmation of inhibitory effect on SPI-1 secretion from small-compound, we engineered *S.* Typhimurium SA strain harboring strep-tag epitope on C-terminus of SipA effector protein. These bacterial strains were grown in a culture medium containing different concentrations of C_24_H_17_ClN_4_O_2_S compound (10, 20, 50, and 100 μM) for 4 h. The bacterial cell culture supernatant was analyzed for SipA-strep-tag protein. Results from Western blotting demonstrated that the compound was concentration-dependent inhibition of SipA effector secretion (Fig. 2C). This observation suggests that the inhibitory effect of the C_24_H_17_ClN_4_O_2_S compound to SPI-1 T3SS appears to occur in a dose-dependent manner.

### C_24_H_17_ClN_4_O_2_S compound significantly blocked the invasion of bacteria into cells

**Fig. 3:**
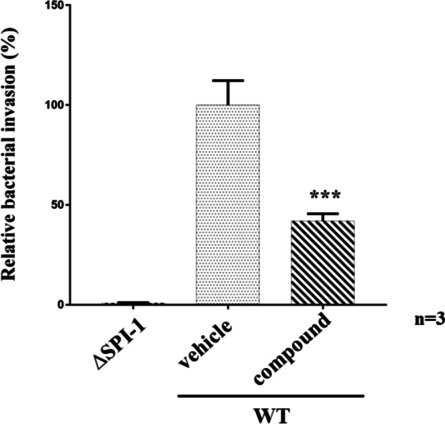
The small molecule decreased the efficiency of bacterial invasion. The standard deviations from three individual experiments are indicated. Asterisks indicate significantly different (*p*<0.05) between wild-type *Salmonella* treatment with or without compound. The SPI-1-negative strain (*invC::kan*^*R*^) was used as control

SipA and SipC effector proteins have been shown to regulate actin cytoskeleton dynamics during the endocytic pathway [25]. The above results showed that the C_24_H_17_ClN_4_O_2_S compound was able to block the secretion of SipA and SipC effector proteins via SPI-1 T3SS. We expected that this compound might affect the ability for invasions into host cells of *S*. Typhimurium. The result from bacterial invasion assay showed that this compound inhibited approximately 50% of invasion by *S.* Typhimurium at 100 μM, which was significantly different (*P*<0.05) from the non-treated *S.* Typhimurium, while T3S-defective (*invC::kan*^*R*^) control strain illustrated low ability to infect into culture cells (Fig. 3). It indicated that the ability of this small molecule to inhibit SipA and SipC secretion impairs the capacity of *S*. Typhimurium to invade host cells.

T3S inhibition effect from C_24_H_17_ClN_4_O_2_S compound did not cause by inactivation of InvC, T3SS ATPase

In T3SS, the ATPase enzyme plays an important role in separation of chaperones and effector proteins before secretion of unfolded effector proteins through needle complex [11]. From above results showed the small molecule, C_24_H_17_ClN_4_O_2_S, had capable of inhibiting the secretion of SPI-1 T3SS effector proteins. We hypothesized that the C_24_H_17_ClN_4_O_2_S compound was able to have inhibitory effect on SP-1 T3SS because it might inhibit the activity of InvC ATPase in *S.* Typhimurium.

**Fig. 4:**
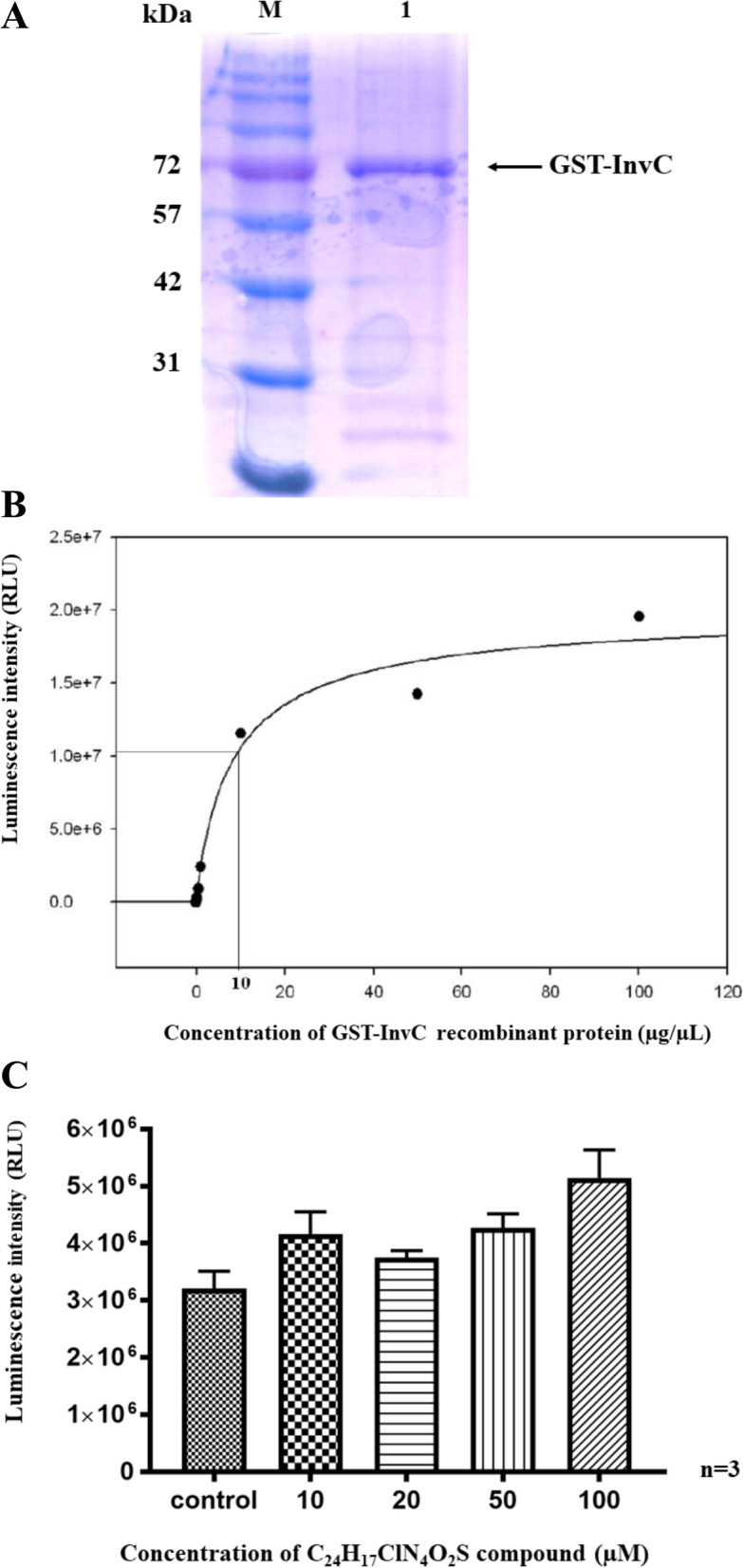
The small-molecule compound did not abolish the activity of InvC ATPase. **A** Purification of soluble GST-InvC fusion protein from bacterial cell lysates. **B** The optimal concentration of InvC ATPase recombinant protein for ATP hydrolytic activity was 10 μg/μL. **C** The compound did not interfere the ATP hydrolysis reaction with InvC ATPase. The data presented from three independent experiments and values were expressed in standard deviations from means. The reaction without inhibitor was used as the control

For the preparation of GST-InvC recombinant protein, *E. coli* BL21 (DE3) strain harboring pGWC recombinant plasmid were cultured under optimal condition (0.5 mM IPTG for 4 h). Bacterial cells were lysed by sonication. The soluble GST-InvC fusion proteins were purified with Glutathione-Agarose resins, then recombinant proteins were eluted with 10-mM glutathione. The result from SDS-PAGE indicated that the elution fraction contains the GST-InvC recombinant proteins (approximately 75 kDa) (Fig. 4A). The concentration of purified protein was about 10 mg/mL.

The optimal amount of InvC ATPase was determined for subsequent ATPase activity assay using curve fitting method from GraphPad Prism sigmoid dose-response software. In this study, the optimal concentration of InvC ATPase for degradation of ATP was 10 μg/μL (Fig. 4B). The previous study reported that a small molecule, C_24_H_17_ClN_4_O_2_S, was able to inhibit T3S ATPase, YscN, the activity of *Y. pestis*. As the result, effector proteins failed to be secreted through T3SS in *Yersinia* [17]. InvC is the ATPase for SPI-1 T3SS and has significant sequence homology with T3S-associated in *Y. pestis*, YscN [11]. We hypothesized that this compound might have inhibitory activity of SPI-1 T3S ATPase, InvC, in *Salmonella*. We determined the inhibition activity of this compound at different concentrations on InvC ATPase using ADP-Glo^TM^ Max Assay. The result showed that concentrations 10, 20, 50, and 100 μM of this compound did not reduce the unit of luminescence intensity compared with the control sample (Fig. 4C). It indicated this compound has no inhibitive effect on the activity of the InvC ATPase enzyme. Therefore, it concluded that the ability to inhibit T3S of this compound did not cause inhibition of InvC ATPase activity.

T3SS transcription factor, InvF, disappeared in C_24_H_17_ClN_4_O_2_S compound-treated bacteria

To further explore the action of the C_24_H_17_ClN_4_O_2_S compound on SPI-1 T3SS, we performed a proteomic analysis for the identification of the inhibition mechanism of this compound on SPI-1 T3SS. As shown in Table 3, results from proteomic profiles illustrated that bacterial cells treated with 100 μM of the compound showed significantly decreased quantification of *S.* Typhimurium Ara-C family regulator of T3SS, InvF, protein. Moreover, the level amount of effector proteins, SipA, and SipC dramatically dropped in compound-treated bacteria. These results provided evidence that the C_24_H_17_ClN_4_O_2_S compound was able to affect the production of multiple SPI-1 effector proteins by reduction of T3SS regulator protein, InvF, level.

**Table 3:** Absolute selected proteins from quantitative proteomic analysis comparison between cellular proteins of *S.* Typhimurium in the presence of vehicle control (DMSO) or 100 μM compound

gi number	Protein name	DMSO-treated	Compound-treated
gi: 52212981	Putative AraC-family regulator of type III secretion system, InvF	5.39963	0
gi: 618634010	Pathogenicity island 1 effector protein, SipA	4.821184	0
gi: 975296	SipC	6.675706	0

## Discussion

Infectious diseases are a major public health problem worldwide. The disease has a high rate of morbidity and mortality compared with non-infectious. Treatment of bacterial infections is necessary to get antibiotics for killing bacteria and patients recovering. However, the frequent occurrence of pathogens that are resistant to traditional antibiotics becomes a great health problem. Therefore, the development of a new antimicrobial drug is required. Antibiotic replacement therapies that target bacterial pathogenesis may be an alternative way to treatment of bacterial infection. The T3SS is one of the most interesting targets for drug discovery. Because this system is the main mechanism for bacterial virulence protein transportation into host cells. Moreover, T3SS has found in gram-negative bacteria that causes disease in humans, such as *Salmonella* [26], *Shigella* [27], *Pseudomonas* [27], enteropathogenic *Escherichia coli* (EPEC) [28], *Vibrio* [27], *Yersinia* [29], and *Chlamydia* [30]. Several compounds from the synthesis or natural product were discovered as T3SS inhibitors [31]. Here, we demonstrated that the small molecule, C_24_H_17_ClN_4_O_2_S, had an inhibitory effect on the secretion of SPI-1 effector proteins, SipA and SipC. Both effector proteins are required for membrane ruffling and invasion of *Salmonella* into host cells. For these reasons, this small molecule decreased the ability to invade host cells of *Salmonella*. Moreover, this compound did not appear to interfere with the growth of bacteria. Thus, the C_24_H_17_ClN_4_O_2_S compound may be a potential candidate for a novel T3SS inhibitor and be less likely to induce the resistance of *Salmonella* to this compound.

The previous study showed that the C_24_H_17_ClN_4_O_2_S compound was capable of inhibiting ATPase activity in *Y. pestis* [17]. The ATPase activity combined with proton motive force is essential to supply energy for T3S [13, 32]. The inactivation of ATPase by the C_24_H_17_ClN_4_O_2_S compound led to inhibit protein secretion through T3SS of *Y. pestis*. Moreover, this compound was also non-toxic to the mammalian cells due to a low potential to target human enzymes [17]. However, in this study, the activity of the SPI-1 T3SS ATPase enzyme treated with this molecule did not decrease. It assumed that this molecule affected T3S ability in *S.* Typhimurium by different pathways. We identified the mechanism of action of this compound on SPI-1 T3SS using proteomic analysis. Protein quantification from mass spectrophotometry indicated that untreated bacteria differed in three SPI-1 T3SS-related proteins from the compound-treated group. Two of them were SPI-1 T3SS effector proteins, SipA and SipC. Another protein was the SPI-1 regulator protein, InvF protein. InvF is a transcriptional activator encoding by SPI-1. This protein is required for the activation of the expression of SPI-1 T3SS effectors encoded both inside and outside of SPI- 1 [33, 34]. The activity of InvF requires SicA, which also encodes within SPI-1 [35, 36]. A recent study showed that InvF/SicA complex could act as a transcription activator for T3SS effector proteins [37]. However, the control of expression of InvF is under control by the central regulator of SPI-1, HilA [38, 39]. The expression of *hilA* is influenced by environmental changes, such as osmolarity, pH, or oxygen tension [23]. Moreover, the expression of the *invF* gene is improved by upregulating FliZ and HilD proteins [40]. The global regulatory system, ArcAB, also promotes the expression of the *invF* gene [41, 42].

**Fig. 5:**
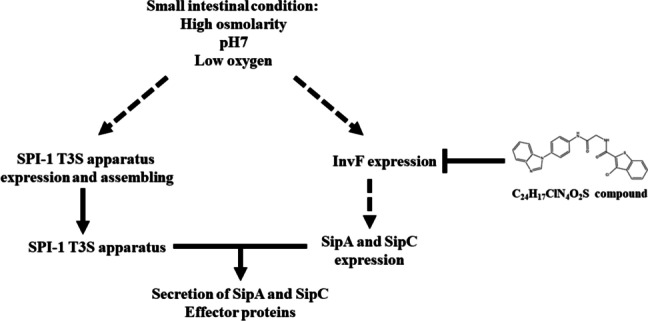
The proposed inhibition pathways of C_24_H_17_ClN_4_O_2_S compound on secretion of SPI-1 effector proteins. The dotted line arrow indicated transcription activation. The blunt ended line indicated inhibition

To date, many T3SS inhibitors from chemical synthesis [18] or natural products [31] have been screened for developing novel anti-virulence agents, such as salicylidene acylhydrazides [43], *N*-phenyl-benzamide [16], caminosides [44], Aurodox [45], and Cytosporone B [46]. The mechanisms of T3SS inhibitors changed in gene expression of T3SS regulator proteins, effector protein, and/or needle apparatus to present their inhibition of T3SS. Interestingly, our results suggested that the C_24_H_17_ClN_4_O_2_S compound may not affect protein for SPI-1 T3SS apparatuses and SPI-1 regulator protein level except for InvF. Therefore, the C_24_H_17_ClN_4_O_2_S compound may influence only protein InvF resulting in to inhibit the secretion of SPI-1 effectors, SipA and SipC (Fig. 5). The mechanism of action of compound affecting InvF will be required to further investigate in the future. The mechanism is supposed to be reducing the transcription of the *invF* gene and increasing the InvF protein degradation or inhibition of InvF binding to the SPI-1 promotor.

## Conclusions

Anti-T3S ATPase in *Yersinia*, C_24_H_17_ClN_4_O_2_S, was identified as anti-T3SS in *Salmonella* in different mechanisms. This compound affects the protein level of SPI-1 regulator protein, InvF, in *Salmonella*. Lacking intracellular InvF protein exerts an inhibitory effect on SPI-1 effector protein secretion. The activities of this compound against *Salmonella* showed that it is possible to be a potential candidate for anti-SPI-1 T3SS substance, likely combined with other agents, to use as a novel anti-salmonellosis agent.

## Data Availability

Not applicable

## Abbreviations

T3SS: Type 3 secretion system
SPI: *Salmonella* pathogenicity island
SCVs: *Salmonella* containing vacuoles
PMF: Proton motive force
IPTG: Isopropyl β-D-1-thiogalactopyranoside
DMSO: Dimethyl sulfoxide
ATCC: American Type Culture Collection
DMEM: Dulbecco’s modified Eagle’s medium
MOI: Multiplicity of infection
LB: Luria-Bertani
GeLC-MS: Gel-enhanced liquid chromatography-mass spectrometry
DTT: Dithiothreitol
ACN: Acetonitrile
FA: Formic acid
EPEC: Enteropathogenic *Escherichia coli*
